# Dynamic Averaging Load Balancing on Cycles

**DOI:** 10.1007/s00453-021-00905-9

**Published:** 2021-12-24

**Authors:** Dan Alistarh, Giorgi Nadiradze, Amirmojtaba Sabour

**Affiliations:** IST Austria, Am Campus 1, 3400 Klosterneuburg, Austria

**Keywords:** Algorithms, Load balancing

## Abstract

We consider the following dynamic load-balancing process: given an underlying graph *G* with *n* nodes, in each step $t\ge 0$, a random edge is chosen, one unit of load is created, and placed at one of the endpoints. In the same step, assuming that loads are arbitrarily divisible, the two nodes *balance* their loads by averaging them. We are interested in the expected gap between the minimum and maximum loads at nodes as the process progresses, and its dependence on *n* and on the graph structure. Peres et al. (Random Struct Algorithms 47(4):760–775, 2015) studied the variant of this process, where the unit of load is placed in the least loaded endpoint of the chosen edge, and the averaging is not performed. In the case of dynamic load balancing on the cycle of length *n* the only known upper bound on the expected gap is of order $\mathcal {O}( n \log n )$, following from the majorization argument due to the same work. In this paper, we leverage the power of averaging and provide an improved upper bound of $\mathcal {O} ( \sqrt{n} \log n )$. We introduce a new potential analysis technique, which enables us to bound the difference in load between *k*-hop neighbors on the cycle, for any $k \le n/2$. We complement this with a “gap covering” argument, which bounds the maximum value of the gap by bounding its value across all possible subsets of a certain structure, and recursively bounding the gaps within each subset. We also show that our analysis can be extended to the specific instance of Harary graphs. On the other hand, we prove that the expected second moment of the gap is lower bounded by $\Omega (n)$. Additionally, we provide experimental evidence that our upper bound on the gap is tight up to a logarithmic factor.

## Introduction

This paper considers balls-into-bins processes where a sequence of *m* weights are placed into *n* bins via some randomized procedure, with the goal of minimizing the load imbalance between the most loaded and the least loaded bin. This family of randomized processes has been used to model several practical allocation problems, such as load-balancing [3, 14, 20], hashing [9], or even relaxed data structures [1, 2].

The classic formulation of this problem is known as *d*-choice process, in each step, a new weight is generated, and is placed in the least loaded of *d* randomly chosen bins. If $d = 1$, then we have the uniform random choice scheme, whose properties are well understood, e.g. [18]. In particular, if we place $m = n$ unit weights into the bins, then it is known that the most loaded bin will have expected $\Theta ( \log n/\log \log n )$ load, whereas if $m = \Omega ( n \log n )$ we have that the expected maximum load is $m/n + \Theta ( \sqrt{ m \log n/n } )$. Seminal work by Azar et al. [3] showed that, if we place *n* unit weights into *n* bins by the *d*-choice process with $d \ge 2$, then, surprisingly, the maximum load is reduced to $\Theta ( \log \log n/\log d)$. A technical tour-de-force by Berenbrink, Czumaj, Steger, and Vöcking [4] extended this result to the “heavily-loaded” case where $m \gg n$, showing that in this case the maximum load is $m/n + \log \log n/\log d + O(1)$ with failure probability at most $1/{\text { poly}} n$. An elegant alternative proof for a slightly weaker version of this result was later provided by Talwar and Wieder [23].

More recently, Peres et al. [17] considered the *graphical* version of this process, where the bins are the vertices of a graph, an *edge* is chosen at every step, and the weight is placed at the less loaded endpoint of the edge, breaking ties arbitrarily. (The reader will notice that the classic 2-choice process corresponds to the case where the graph is a clique.) The authors focus on the evolution of the gap between the highest and lowest loaded bins, showing that, for graphs of $\beta$-edge-expansion [17], this gap is $O( \log n/\beta )$, with probability $1 - 1/{\text {poly}} n$.

Another closely related line of work considers *static load-balancing* processes, where each node in a graph starts with an arbitrary initial load, and the endpoints average their current loads at each step. Note that load balancing schemes which are commonly used in this setting are usually more involved than simply averaging the loads of the endpoints of the randomly chosen node, but the tools used in their analysis are still applicable to the static version of our process. To analyze such processes, it is common to map the process to a Markov chain and analyse its convergence [6, 10, 12], or derive an upper bound on a potential function which captures the discrepancy between the loads [5]. In both cases, the gap between the highest and lowest loaded bins can be characterized by the spectral gap of the graph [7, 11, 19, 21, 22].

By contrast to these two lines of previous work, in this paper we consider a graphical load balancing in the *dynamic* case, where weights arrive at each step rather than being statically allocated initially, but we allow balancing via *continuous averaging*, i.e. the resulting weights after the balancing step equal the average of the sum of the weights of the two nodes prior to balancing. Thus, our averaging step is more powerful relative to *d*-choice or static averaging, but it is applied in the more challenging *dynamic* scenario.

We will focus on the gap in the dynamic case on graphs of low expansion, specifically on cycles. In [17], it is shown that, in this case (but without averaging), the gap is $O(n \log n)$ both in expectation and with high probability. The techniques used in [17] imply that the averaging of the loads does not worsen the gap in expectation, our aim is to show that it actually helps to reduce it. Also, directly applying the tools from the static process to the dynamic one results in the upper bound which is larger than $O(n \log n)$. Upper bounding the gap for cycle graphs is known to be a challenging open problem [16]. As suggested in [17], to deal with the cycle case, there is a need for a new approach, which takes the structure of the load balancing graph into account.

### Contribution

In this paper, we address this question for the case where averaging is performed on a cycle graph. Let *Gap*(*t*) be a difference between highest and lowest loads of the nodes at time step *t*. We provide the upper bound on the gap in the dynamic, heavily-loaded case, via a new potential argument. More formally, for any $t>0$, we show that for a cycle graph with *n* vertices:

1 $$\begin{aligned} \mathbb {E}[Gap(t)] = O(\sqrt{n} \log (n)). \end{aligned}$$

We show that our technique can be used to upper bound the gap for the 4-connected Harary Graph. We complement this result with a lower bound of $\Omega ( n )$ on $\mathbb {E}[(Gap(t))^2]$. Further, we provide experimental evidence that the gap is of order $\Theta (\sqrt{n})$, making our upper bound accurate up to a $\log {n}$ factor. Our results extend to the case where the load generated at each node is *weighted* according to some distribution whose second moment is bounded. Formally, we allow our input to come from any distribution *W*, such that $\mathbb {E}[W^2] \le M^2$, for some $M > 0$.

### Technical Overview

Our upper bound result is based on two main ideas. The first introduces a new parametrized hop-potential function, which measures the squared difference in load between any *k*-hop neighbors on the graph, where $k \ge 1$ is a fixed hop parameter. Let $G = (V, E)$ be our input graph, where $V=\{1,2,\ldots ,n\}$. Throughout the paper, for any $1 \le i \le n$ we assume that the nodes $i+n$ and $i-n$ are the same as the node *i*. Let $x_i(t)$ be the load of node *i* at step *t*. Then, we define the *k*-hop potential as:

$$\begin{aligned} \phi _k(t) = \sum _{i=1}^{n} (x_i(t) - x_{i+k}(t))^2. \end{aligned}$$

The first technical step in the proof is to understand the expected (“steady-state”) value of the *k*-hop potential. We show that, in expectation, the *k*-hop potential has a recursive structure. While the expected values of *k*-hop potentials cannot be computed precisely, we can isolate upper and lower bounds on their values for cycles. In particular, for the *k*-hop potential on an *n*-cycle, we prove the following bound:

2 $$\begin{aligned} \mathbb {E}[\phi _k(t)] \le k(n-k)-1,\quad \forall k \ge 1. \end{aligned}$$

In the second technical step, we shift gears, aiming to bound the *maximum possible value* of the gap between any two nodes, leveraging the fact that we understand the hop potential for any $k \ge 1$. We achieve this via a “gap covering” technique, which characterizes the maximum value of the gap across all possible subsets of a certain type.

More precisely, in the case of a cycle of length $n = 2^m$, for each node *i* and hop count *k*, we define the set family $A_{k}^i$ to be formed of nodes $\{i, i+2^{m-k}, i + 2\times 2^{m-k}, i + 3\times 2^{m-k}, \dots \}$ (since we are on a cycle, $i = i + 2^{m - k} 2^k$). Then for any $1 \le k \le m$, we will have

3 $$\begin{aligned} \sum _{i=1}^n Gap_{A_{k}^i}(t) \le \sum _{i=1}^n Gap_{A_{k-1}^i}(t)+\frac{n}{\sqrt{2^{m-k}}}\sqrt{\phi _{2^{m-k}}(t)}, \end{aligned}$$

where $Gap_{X}(t)$ is the maximal gap inside the set *X* at time *t*. Intuitively, this result allows us recursively characterize the gap value at various “resolutions” across the graph.

Finally, we notice that we can “cover” the gap between *any* two nodes by carefully unwinding the recursion in the above inequality, considering all possible subsets of a well-chosen structure, and recursively bounding the gaps within each subset (this step is particularly delicate in the case where *n* is not a power of two, please see Sect. 5). We obtain that

4 $$\begin{aligned} \mathbb {E}[Gap(t)] = O(\sqrt{n} \log (n)), \end{aligned}$$

as claimed. The logarithmic slack is caused by the second term on the right-hand-side of (2). We note that this technique extends to the case where inserted items are *weighted*, where the weights are coming from some distribution of bounded second moment.

### Lower Bound

It is interesting to ask whether this upper bound is tight. To examine this question, we revisit the recursive structure of the *k*-hop potential, which we used to obtain the upper bound in Eq. (3). We can leverage this structure to obtain a *lower bound* on the expected *k*-hop potential as well. Starting from this lower bound, we can turn the upper bound argument “inside out,” to obtain a linear lower bound on the *expected squared gap*:

5 $$\begin{aligned} \mathbb {E}[Gap(t)^2] = \Omega (n). \end{aligned}$$

We conjecture that both upper and lower bounds on the expected gap are of order $O(\sqrt{n})$ (given that $E[W^2]$ is constant), and examine this claim empirically in Sect. 6.

### Extensions and Discussion

We believe that the analysis template we described above is general, and can be extended to other graph families, such as regular expanders. Here, we focus on obtaining tight bounds on the gap for cycles, which is technically non-trivial, and leave the extensions for other graph families as future work. To substantiate our generality claim, we exhibit an application of our analysis technique to the specific instance of Harary graphs [13] in Sect. 7. More precisely, we provide the upper bound on a gap for a graph on *n* vertices, where each vertex *i* is connected with edges to vertices $i-2,i-1,i+1$ and $i+2$.

We discuss the relation between our results and bounds for the graphical power-of-two process on a cycle [17] in Sect. 8.

### Related Work

As we have already discussed broad background, we will now mainly focus on the technical differences from previous work. As stated, we are the first to specifically consider the *dynamic* case for *continuous averaging* on cycles. The *static* case has been studied both with *continuous averaging* [5–7, 10–12, 19, 22] and *discrete averaging* [21]. However, their techniques would not apply (or would result in a worse bound) in our case, since we consider that weights would be introduced *dynamically*, during the processes’ execution.

To our knowledge, the only non-trivial upper bound on the gap of the process we consider which would follow from previous work is of $\mathcal {O}( n \log n )$, by the potential analysis of [17]: they consider 2-choice load balancing, and one can re-do their potential analysis for (continuous) averaging load balancing, yielding the same bounds. (Specifically, one can use the same definition of the potential $\Gamma (t)$ as in this reference, and will obtain the same upper bounds, since the pairwise load balancing we consider has slightly stronger guarantees.) However, as our bounds show, the resulting analysis is quite loose in the case of cycle graphs, yielding an $\Omega ( \sqrt{n} )$ gap between the bounds yielded by these techniques. Technically, this is a consequence of the majorization technique used in [17], which links dynamic averaging on the cycle with a very weak form of averaging on the clique. Reference [8] studies the performance differences between various load-balancing techniques; specifically, it shows that continuous averaging cannot improve the bound on the gap relative to *d*-choice on the clique. Unfortunately, this result does not seem to extend to arbitrary graphs.

Our potential analysis is substantially different from that of [17], as they track a sum of exponential potentials across the entire graph. By contrast, our analysis tracks the squared load differences between *k*-hop neighbors, establishing recurrences between these potentials. We note that this is also different from the usual square potentials used for analyzing averaging load balancing, e.g. [15], which usually compare against the *global mean*, as opposed to pairwise potential differences. Our approach is also different from the classic analyses of e.g. [3], which perform probabilistic induction on the number of bins at a given load, assuming a clique.

Generally, our technique can be seen as performing the induction needed to bound the gap not on the bin loads, as is common in previous work, e.g. [3], but *over the topology of the graph*. This approach is natural, since we wish to obtain tight, topology-specific bounds, but we believe we are the first to propose and analyze it successfully.

## Averaging on the Cycle: Upper Bounding the Gap

### Preliminaries

We consider a cycle graph $G = (V, E)$ where $V=\{1, 2, \ldots , n\}$, such that each node *i* is connected to its left and right neighbors, $i - 1$ and $i + 1$ (recall that for any $1 \le i \le n$ the nodes $i+n$ and $i-n$ are the same as the node *i*).

We consider a stochastic process following real time $t \ge 0$, in which, at each step $t+1$, a ball of weight $w(t)\ge 0$ is generated from a same distribution *W*. We associate a real-valued *load* $x_i(t)$ with each node *i* ($x_i(t)$ is the value after *t* steps). Initially, we have that $x_i(0)=0$ for every node *i*. At step $t+1$, an edge $(i, i + 1)$ is chosen uniformly at random, and the two endpoint nodes update their weights as follows:

$$\begin{aligned} x_i(t + 1) = x_{i + 1} (t + 1) = \frac{x_{i}(t) + x_{i+1}(t) + w(t)}{2}. \\ \end{aligned}$$

We will assume that the second moment of the distribution *W* is bounded. That is: $E[W^2] \le M^2$, for some $M>0$. For simplicity, we will assume that weights are normalized by *M*. This gives us that $\mathbb {E}[W^2] \le 1$.

Let $X(t)=(x_1(t), x_2(t), \ldots , x_n(t))$ be the vector of the bin weights after step *t*. First, we define the following potential functions:

$$\begin{aligned} \forall k \in \{1, 2, \dots , n-1\}: \phi _k(t) :=\sum _{i=1}^{n} (x_i(t) - x_{i+k}(t))^2. \end{aligned}$$

Notice that for every $1 \le i \le n$, we have that $\phi _i(t)=\phi _{n-i}(t)$. We want to analyze what is the value of these functions in expectation after an additional ball is thrown, for a given load vector *X*(*t*).

We start with $\phi _1(t+1)$:

6 $$\begin{aligned} \mathbb {E}[\phi _1(t&+1) | X(t),w(t)] = \sum _{i=1}^{n} \frac{1}{n} \Bigg (\Big (\frac{x_{i}(t) + x_{i+1}(t) + w(t)}{2} - x_{i+2}(t)\Big )^2 \nonumber \\&\quad \quad \quad \quad \quad \quad \quad \quad \quad \quad \quad \quad + \Big (\frac{x_{i}(t) + x_{i+1}(t) + w(t)}{2} - x_{i-1}(t)\Big )^2 \nonumber \\&\quad \quad \quad \quad \quad \quad \quad \quad \quad \quad \quad \quad + \sum _{j \ne i-1, i, i+1} (x_j(t) - x_{j+1}(t))^2 \Bigg ). \end{aligned}$$

Notice that:

7 $$\begin{aligned} \sum _{i=1}^{n} \sum _{j \ne i-1, i, i+1} (x_j(t) - x_{j+1}(t))^2=(n-3) \phi _1(t). \end{aligned}$$

Hence we need to bound the remaining terms:

$$\begin{aligned} \sum _{i=1}^n&\Bigg ( \Big (\frac{x_{i}(t) + x_{i+1}(t) + w(t)}{2} - x_{i+2}(t)\Big )^2 + \Big (\frac{x_{i}(t) + x_{i+1}(t) + w(t)}{2} - x_{i-1}(t)\Big )^2\Bigg ) \\&=\sum _{i=1}^n \Bigg (\frac{2x_i(t)^2 + 2x_{i+1}(t)^2 + 2w(t)^2 + 4x_i(t)x_{i+1}(t)}{4} \\&\qquad + x_{i+2}(t)^2 + x_{i-1}(t)^2 - (x_i(t) + x_{i+1}(t))(x_{i-1}(t) + x_{i+2}(t))\Bigg ). \end{aligned}$$

where we used the fact that terms, which are linear in *w*(*t*), cancel out.

The right side of the above equation can be rewritten as:

$$\begin{aligned}&\sum _{i=1}^n \frac{x_i(t)^2}{2}+\sum _{i=1}^n \frac{x_{i+1}(t)^2}{2} +\sum _{i=1}^n x_{i+2}(t)^2 +\sum _{i=1}^n x_{i-1}(t)^2 \\&\qquad \quad +\sum _{i=1}^n x_i(t)x_{i+1}(t)-\sum _{i=1}^n x_i(t)x_{i-1}(t)-\sum _{i=1}^n x_{i+1}(t)x_{i+2}(t) \\&\qquad \quad -\sum _{i=1}^n x_i(t)x_{i+2}(t)-\sum _{i=1}^n x_{i+1}(t)x_{i-1}(t) +\frac{nw(t)^2}{2} \\&\quad = 3\sum _{i=1}^n x_{i}(t)^2-\sum _{i=1}^n x_i(t)x_{i+1}(t)- 2\sum _{i=1}^n x_i(t)x_{i+2}(t)+\frac{nw(t)^2}{2} \\&\quad = \sum _{i=1}^n \frac{\Big (x_i(t)-x_{i+1}(t)\Big )^2}{2}+\sum _{i=1}^n \Big (x_i(t)-x_{i+2}(t)\Big )^2+\frac{nw(t)^2}{2} \\&\quad = \frac{\phi _1(t)}{2}+\phi _2(t)+ \frac{nw(t)^2}{2}. \end{aligned}$$

By using the above equation and Eq. (7) in Eq. (6) we get that

$$\begin{aligned} \mathbb {E}[\phi _1(t&+1) | X(t),w(t)]=\frac{n-2}{n}\phi _1(t)+\frac{1}{2}\Big (w(t)^2-\frac{\phi _1(t)}{n}\Big )+\frac{\phi _2(t)}{n}. \end{aligned}$$

Now, we proceed with calculating the expected value of $\phi _k(t+1)$,

for $$2 \le k \le \lfloor n/2 \rfloor $$:

$$\begin{aligned} \mathbb {E}[\phi _k(t&+1) | X(t),w(t)] = \sum _{i=1}^{n} \frac{1}{n} \Bigg (\Big (\frac{x_{i}(t) + x_{i+1}(t) + w(t)}{2} - x_{i-k}(t)\Big )^2 \\&\quad \quad \quad \quad \quad \quad \quad \quad \quad \quad \quad + \Big (\frac{x_{i}(t) + x_{i+1}(t) + w(t)}{2} - x_{i+1-k}(t)\Big )^2 \\&\quad \quad \quad \quad \quad \quad \quad \quad \quad \quad \quad + \Big (\frac{x_{i}(t) + x_{i+1}(t) + w(t)}{2} - x_{i+k}(t)\Big )^2 \\&\quad \quad \quad \quad \quad \quad \quad \quad \quad \quad \quad + \Big (\frac{x_{i}(t) + x_{i+1}(t) + w(t)}{2} - x_{i+1+k}(t)\Big )^2 \\&\quad \quad \quad \quad \quad \quad \quad \quad \quad \quad \quad + \sum _{j \ne i-k, i+1, i, i+1} (x_j(t) - x_{j+k}(t))^2 \Bigg ). \end{aligned}$$

Notice that:

$$\begin{aligned} \frac{1}{n}\sum _{i=1}^n \sum _{j \ne i-k, i+1-k, i+k, i+1+k} (x_j(t) - x_{j+k}(t))^2=\frac{n-4}{n} \phi _k(t). \end{aligned}$$

In the similar way as for $\phi _1(t)$ the remaining terms can be rewritten as:

$$\begin{aligned}&\sum _{i=1}^{n}\frac{1}{n} \Bigg (x_i(t)^2 + x_{i+1}(t)^2 + w(t)^2 + 2x_i(t)x_{i+1}(t) + 4x_i^2(t) \\&\quad \quad \quad \quad - \Big (x_i(t) + x_{i+1}(t)\Big )\Big ( x_{i+k}(t) + x_{i+k+1}(t) + x_{i-k}(t) + x_{i-k+1}(t)\Big )\Bigg ) \\&= \frac{1}{n} \Bigg (nw(t)^2 + 6\sum _{i=1}^n x_i(t)^2 + 2\sum _{i=1}^n x_i(t)x_{i+1}(t) - 4\sum _{i=1}^n x_i(t)x_{i+k}(t) \\&\quad \quad \quad \quad \quad \quad \quad \quad \quad - 2\sum _{i=1}^n x_i(t)x_{i+k+1}(t) - 2\sum _{i=1}^n x_i(t)x_{i+k-1}(t)\Bigg ) \\&= \frac{2}{n} \phi _k(t) + (w(t)^2 - \frac{\phi _1(t)}{n}) + \frac{\phi _{k+1}(t)}{n} + \frac{\phi _{k-1}(t)}{n}. \end{aligned}$$

Hence, we get that:

$$\begin{aligned} \mathbb {E}[\phi _k(t&+1) | X(t),w(t)] = \frac{n-2}{n} \phi _k(t) + (w(t)^2 - \frac{\phi _1(t)}{n}) + \frac{\phi _{k+1}(t)}{n} + \frac{\phi _{k-1}(t)}{n}. \end{aligned}$$

If we remove conditioning on *w*(*t*) (note that $\mathbb {E}[w(t)^2]=\mathbb {E}[W^2]$) and express these equations for

$\phi _1(t+1), \phi _2(t+1), \dots , \phi _{n-1}(t+1)$ (recall that $\phi _k(t)=\phi _{n-k}(t)$), we get:

8 $$\begin{aligned} {\left\{ \begin{array}{ll} \mathbb {E}[\phi _1(t+1)|X(t)] = (\frac{n-2}{n})\phi _1(t) + \frac{1}{2}(\mathbb {E}[W^2] - \frac{\phi _1(t)}{n}) + \frac{\phi _2(t)}{n}. \\ \mathbb {E}[\phi _2(t+1)|X(t)] = (\frac{n-2}{n})\phi _2(t) + (\mathbb {E}[W^2] - \frac{\phi _1(t)}{n}) + \frac{\phi _1(t)}{n} + \frac{\phi _3(t)}{n}. \\ \dots \\ \mathbb {E}[\phi _{\lfloor \frac{n}{2} \rfloor }(t+1)|X(t)] = (\frac{n-2}{n})\phi _{\lfloor \frac{n}{2} \rfloor }(t) + (\mathbb {E}[W^2] - \frac{\phi _1(t)}{n}) \\ \quad \quad \quad \quad \quad \quad \quad \quad \quad + \frac{\phi _{\lfloor \frac{n}{2} \rfloor - 1}(t)}{n} + \frac{\phi _{\lfloor \frac{n}{2} \rfloor + 1}(t)}{n}. \\ \dots \\ \mathbb {E}[\phi _{n-2}(t+1)|X(t)] = (\frac{n-2}{n})\phi _{n-2}(t) \\ \quad \quad \quad \quad \quad \quad \quad \quad \quad + (\mathbb {E}[W^2] - \frac{\phi _1(t)}{n}) + \frac{\phi _{n-3}(t)}{n} + \frac{\phi _{n-1}(t)}{n}. \\ \mathbb {E}[\phi _{n-1}(t+1)|X(t)] = (\frac{n-2}{n})\phi _{n-1}(t) + \frac{1}{2}(\mathbb {E}[W^2] - \frac{\phi _1(t)}{n}) + \frac{\phi _{n-2}(t)}{n}. \\ \end{array}\right. } \end{aligned}$$

Using the above equations we can prove the following:

#### Lemma 1

For every $t \ge 0$ and $1 \le k \le n-1$, we have that

9 $$\begin{aligned} \mathbb {E}[\phi _k(t)] \le (k(n-k)-1) \mathbb {E}[W^2] \le k(n-k)-1. \end{aligned}$$

#### Proof

Let $\Phi (t)=(\phi _1(t), \phi _2(t), \ldots , \phi _{n-1}(t))$ be the vector of values of our potentials at time step *t* and let $Y=(y_1, y_2, \ldots , y_{n-1})$, be the vector containing our desired upper bounds for each potential. That is: for each $1 \le i \le n-1$, we have that $y_i=(i(n-i)-1)\mathbb {E}[W^2]$.

An interesting and easily checkable thing about the vector *Y* is that

10 $$\begin{aligned} \mathbb {E}[\Phi (t+1)|\Phi (t)=Y]=Y. \end{aligned}$$

Next, consider the vector $Z(t)=(z_1(t), z_2(t), \ldots z_{n-1}(t))=Y-\Phi (t)$. Our goal is to show that for every step *t* and coordinate *i*, $\mathbb {E}[z_i(t)]\ge 0$.

We have that

$$\begin{aligned} \mathbb {E}[&z_1(t+1)|X(t)]=y_1-\mathbb {E}[\phi _1(t+1)|X(t)] \\&= (\frac{n-2}{n})y_1 + \frac{1}{2}(\mathbb {E}[W^2] - \frac{y_1}{n}) + \frac{y_2}{n} \\&\quad \quad \quad \quad \quad - \Bigg ((\frac{n-2}{n})\phi _1(t) + \frac{1}{2}(\mathbb {E}[W^2] - \frac{\phi _1(t)}{n}) + \frac{\phi _2(t)}{n} \Bigg ) \\&= (\frac{n-2}{n})z_1(t) - \frac{z_1(t)}{2n} + \frac{z_2(t)}{n}. \end{aligned}$$

and for $2 \le i \le \lfloor \frac{n}{2} \rfloor$, we have that

$$\begin{aligned} \mathbb {E}[z_i(t&+1)|X(t)]= (\frac{n-2}{n})z_i(t) - \frac{z_1(t)}{n} + \frac{z_{i+1}(t)}{n}+ \frac{z_{i-1}(t)}{n}. \end{aligned}$$

Hence we get the following equations (recall that $z_i(t)=z_{n-i}(t)$):

11 $$\begin{aligned} {\left\{ \begin{array}{ll} n \times \mathbb {E}[z_1(t+1)|X(t)] = (n - 2 - \frac{1}{2}) z_1(t) + z_2(t). \\ n \times \mathbb {E}[z_2(t+1)|X(t)] = -z_1(t) + z_1(t) + (n - 2) z_2(t) + z_3(t). \\ n \times \mathbb {E}[z_3(t+1)|X(t)] = -z_1(t) + z_2(t) + (n - 2) z_3(t) + z_4(t).\\ \dots \\ n \times \mathbb {E}[z_{\lfloor \frac{n}{2}\rfloor }(t+1)|X(t)] = -z_1(t) + z_{\lfloor \frac{n}{2}\rfloor -1}(t) + (n-2) z_{\lfloor \frac{n}{2}\rfloor }(t) + z_{\lfloor \frac{n}{2}\rfloor +1}(t). \end{array}\right. } \end{aligned}$$

Next, using induction on *t*, we show that for every $t \ge 0$

12 $$\begin{aligned} 0 \le \mathbb {E}[z_1(t)] \le \mathbb {E}[z_2(t)] \le \cdots \le \mathbb {E}[z_{\lfloor \frac{n}{2}\rfloor }(t)]. \end{aligned}$$

The base case holds trivially since $Z(0)=Y$. For the induction step, assume that

$0 \le \mathbb {E}[z_1(t)] \le \mathbb {E}[z_2(t)] \le \cdots \le \mathbb {E}[z_{\lfloor \frac{n}{2}\rfloor }(t)]$. First, we have that

$$\begin{aligned} n\mathbb {E}[z_1(t+1)]=n\mathbb {E}_{X(t)}[\mathbb {E}[z_1(t+1)|X(t)]]=(n - 2 - \frac{1}{2}) \mathbb {E}[z_1(t)] + \mathbb {E}[z_2(t)] \ge 0. \end{aligned}$$

Additionally, we have that:

$$\begin{aligned} n\mathbb {E}[z_1(t+1)]&=(n - 2 - \frac{1}{2}) \mathbb {E}[z_1(t)] + \mathbb {E}[z_2(t)] \le (n - 2) \mathbb {E}[z_1(t)] + \mathbb {E}[z_2(t)] \\&\le (n - 2) \mathbb {E}[z_2(t)] + \mathbb {E}[z_3(t)]=n\mathbb {E}[z_2(t+1)]. \end{aligned}$$

For $2 \le i \le \lfloor \frac{n}{2}\rfloor -2$, we have that

$$\begin{aligned} n\mathbb {E}[z_i(t+1)]&=-\mathbb {E}[z_1(t)]+\mathbb {E}[z_{i-1}(t)]+(n - 2) \mathbb {E}[z_i(t)] + \mathbb {E}[z_{i+1}(t)] \\ {}&\le -\mathbb {E}[z_1(t)]+\mathbb {E}[z_{i}(t)]+(n - 2) \mathbb {E}[z_{i+1}(t)] + \mathbb {E}[z_{i+2}(t)] \\ {}&= n\mathbb {E}[z_{i+1}(t+1)]. \end{aligned}$$

Next, observe that by our assumption:

$\mathbb {E}[z_{\lfloor \frac{n}{2}\rfloor +1}(t)]= \mathbb {E}[z_{\lceil \frac{n}{2}\rceil -1}(t)]\ge \mathbb {E}[z_{\lfloor \frac{n}{2}\rfloor -2}(t)]$. Finally, by using this observation we get that

$$\begin{aligned} n&\mathbb {E}[z_{\lfloor \frac{n}{2}\rfloor -1}(t+1)] =-\mathbb {E}[z_1(t)]+\mathbb {E}[z_{\lfloor \frac{n}{2}\rfloor -2}(t)]+(n - 2) \mathbb {E}[z_{\lfloor \frac{n}{2}\rfloor -1}(t)] + \mathbb {E}[z_{\lfloor \frac{n}{2}\rfloor }(t)] \\ {}&\le -\mathbb {E}[z_1(t)]+\mathbb {E}[z_{\lfloor \frac{n}{2}\rfloor +1}(t)]+\mathbb {E}[z_{\lfloor \frac{n}{2}\rfloor -1}(t)] + (n - 3) \mathbb {E}[z_{\lfloor \frac{n}{2}\rfloor -1}(t)]+\mathbb {E}[z_{\lfloor \frac{n}{2}\rfloor }(t)] \\&\le -\mathbb {E}[z_1(t)]+\mathbb {E}[z_{\lfloor \frac{n}{2}\rfloor +1}(t)]+\mathbb {E}[z_{\lfloor \frac{n}{2}\rfloor -1}(t)]+(n - 2)\mathbb {E}[z_{\lfloor \frac{n}{2}\rfloor }(t)] \\&= n\mathbb {E}[z_{\lfloor \frac{n}{2}\rfloor }(t+1)]. \end{aligned}$$

This completes the proof of the lemma. $\square$

## Upper Bound on the Gap for $n=2^m$

In this section we upper bound a gap in expectation for the case when $n=2^m$. is quite technical but not necessarily more interesting, and is provided in the Sect. 5.

We begin with some definitions. For a set $A \subseteq \{1, 2, \dots , n\}$, let

$$\begin{aligned} Gap_A(t) = \max _{i \in A} x_i(t) - \min _{i \in A} x_i(t). \end{aligned}$$

Also, let $A_{k}^i$ be $\{i, i+2^{m-k}, i + 2\times 2^{m-k}, i + 3\times 2^{m-k}, \dots \}$ (Notice that $i=i+2^{m-k}2^k$). Our proof works as follows: for each $1 \le i \le n$ and $1 \le k \le m$, we look at the vertices given by the sets $A_{k-1}^i$ and $A_{k-1}^{i+2^{m-k}}$ and try to characterise the gap after we merge those sets (note that this will give us the gap for the set $A_{k}^i=A_{k-1}^i \cup A_{k-1}^{i+2^{m-k}}$). Using this result, we are able to show that $\sum _{i=1}^n Gap_{A^i_{k}}(t)$ is upper bounded by $\sum _{i=1}^n Gap_{A_{k-1}^i}(t)$ plus *n* times maximum load difference between vertices at hop distance $2^{m-k}$. Next, we use $2^{m-k}$ hop distance potential $\phi _{2^{m-k}}(t)$ to upper bound maximum load difference between the vertices at hop distance $2^{m-k}$. By summing up the derived inequality for $k=1$ to *m*, we are able to upper bound $\sum _{i=1}^n Gap_{A^i_{m}}(t)$ in terms of $\sum _{i=1}^n Gap_{A^i_{0}}(t)$ and $\sum _{k=1}^{m} \phi _{2^{m-k}}(t)$. Notice that by our definitions, for each *i*, $Gap_{A^i_{0}}(t)=0$ ($A_0^i$ contains only vertex *i*) and $Gap_{A^i_{m}}(t)=Gap(t)$ ($A^{i}_m$ contains all vertices). Hence, what is left is to use the upper bounds for the hop distance potentials, which we derived in the previous section.

We start by proving the following useful lemma.

### Lemma 2

For any $1 \le i \le n$ and $1 \le k \le m$, we have that

13 $$\begin{aligned} 2Gap_{A_{k}^i}(t) \le 2 \max _{j \in A_{k}^i}|x_j(t) - x_{j+2^{m-k}}(t)| + Gap_{A_{k-1}^{i+2^{m-k}}}(t)+ Gap_{A_{k-1}^i}(t).\nonumber \\ \end{aligned}$$

### Proof

Fix vertex *i*. Recall that $A_{k}^i=A_{k-1}^i \cup A_{k-1}^{i+2^{m-k}}$.

Let $u={{\,\mathrm{arg\,max}\,}}_{j \in A_{k}^i} x_j(t)$ and let $v ={{\,\mathrm{arg\,min}\,}}_{j \in A_{k}^i} x_j(t)$. We consider several cases on the membership of nodes *u* and *v*, and bound the gap in each one:

**Case 1** $u \in A_{k-1}^{i}$ and $v \in A_{k-1}^{i}$. Then $Gap_{A_{k}^i}(t)=Gap_{A_{k-1}^i}(t)$ and we have that

$$\begin{aligned} Gap_{A_{k}^i}(t)&= |x_u(t) - x_v(t)| \\&\le |x_{u+2^{m-k}}(t) - x_{u}(t)| + |x_{v+2^{m-k}}(t) - x_{v}(t)| \\&\quad \quad \quad \quad \quad \quad \quad \quad \quad \quad + |x_{u+2^{m-k}}(t) - x_{v+2^{m-k}}(t)| \\&\le |x_{u+2^{m-k}}(t) - x_{u}(t)| + |x_{v+2^{m-k}}(t) - x_{v}(t)| \\&\quad \quad \quad \quad \quad \quad \quad \quad \quad \quad + Gap_{A_{k-1}^{i+2^{m-k}}}(t) \\&\le 2 \max _{j \in A_{k}^i}|x_j(t) - x_{j+2^{m-k}}(t)| + Gap_{A_{k-1}^{i+2^{m-k}}}(t), \end{aligned}$$

where we used the fact that both $u+2^{m-k}$ and $v+2^{m-k}$ belong to $A_{k-1}^{i+2^{m-k}}$. This gives us that

14 $$\begin{aligned} 2Gap_{A_{k}^i}(t) \le 2 \max _{j \in A_{k}^i}|x_j(t) - x_{j+2^{m-k}}(t)| + Gap_{A_{k-1}^{i+2^{m-k}}}(t)+ Gap_{A_{k-1}^i}(t). \end{aligned}$$

**Case 2** $u \in A_{k-1}^{i}$ and $v \in A_{k-1}^{i+2^{m-k}}$. Then we have that:

$$\begin{aligned} Gap_{A_{k}^i}(t)&= |x_u(t) - x_v(t)| \le |x_u(t) - x_{v + 2^{m-k}}(t)| + |x_{v+2^{m-k}}(t) - x_v(t)| \\&\le Gap_{A_{k-1}^{i}}(t) + \max _{j \in A_{k}^i}(|x_j(t) - x_{j+2^{m-k}}(t)|) \end{aligned}$$

and

$$\begin{aligned} Gap_{A_{k}^i}(t)&= |x_u(t) - x_v(t)| \le |x_u(t) - x_{u + 2^{m-k}}(t)| + |x_{u+2^{m-k}}(t) - x_v(t)| \\&\le Gap_{A_{k-1}^{i+2^{m-k}}}(t) + \max _{j \in A_{k}^i}(|x_j(t) - x_{j+2^{m-k}}(t)|), \end{aligned}$$

where we used $v+2^{m-k} \in A_{k-1}^i$ and $u+2^{m-k} \in A_{k-1}^{i+2^{m-k}}$. Hence, we again get that

15 $$\begin{aligned} 2Gap_{A_{k}^i}(t) \le 2 \max _{j \in A_{k}^i}|x_j(t) - x_{j+2^{m-k}}(t)| + Gap_{A_{k-1}^{i+2^{m-k}}}(t)+ Gap_{A_{k-1}^i}(t). \end{aligned}$$

**Case 3** $u \in A_{k-1}^{i+2^{m-k}}$ and $v \in A_{k-1}^{i+2^{m-k}}$, is similar to Case 1.

**Case 4** $v \in A_{k-1}^{i}$ and $u \in A_{k-1}^{i+2^{m-k}}$, is similar to Case 2.

$$\square $$

Next, we upper bound the quantity $\sum _{i=1}^n \max _{j \in A_{k}^i}|x_j(t) - x_{j+2^{m-k}}(t)|$.

### Lemma 3

For any $$1 \le k \le m$$:

16 $$\begin{aligned} \sum _{i=1}^n \max _{j \in A_{k}^i}|x_j(t) - x_{j+2^{m-k}}(t)| \le \frac{n}{\sqrt{2^{m-k}}} \sqrt{\phi _{2^{m-k}}(t)}. \end{aligned}$$

### Proof

Notice that for any *i* and $i' \in A_k^i$, we have that $A_k^i=A_k^{i'}$,

hence $\max _{j \in A_{k}^i}|x_j(t) - x_{j+2^{m-k}}(t)| = \max _{j \in A_{k}^{i'}}|x_j(t) - x_{j+2^{m-k}}(t)|$ and this means that

$$\begin{aligned} \sum _{i=1}^n \max _{j \in A_{k}^i}|x_j(t)&- x_{j+2^{m-k}}(t)| = \frac{n}{2^{m-k}} \sum _{i=1}^{2^{m-k}} \max _{j \in A_{k}^{i}}|x_j(t) - x_{j+2^{m-k}}(t)| \\&\overset{Cauchy{-}Schwarz}{\le } \frac{n}{2^{m-k}}\sqrt{2^{m-k}} \sqrt{\sum _{i=1}^{2^{m-k}} \max _{j \in A_{k}^{i}}|x_j(t) - x_{j+2^{m-k}}(t)|^2} \\&\le \frac{n}{2^{m-k}}\sqrt{2^{m-k}} \sqrt{\sum _{j=1}^n |x_j(t) - x_{j+2^{m-k}}(t)|^2}\\ {}&=\frac{n}{\sqrt{2^{m-k}}} \sqrt{\phi _{2^{m-k}}(t)}, \end{aligned}$$

where in the last inequality we used a fact that sets $A_k^1, A_k^2, \ldots , A_k^{2^{m-k}}$ are disjoint. $\square$

Finally, using the above lemmas we can upper bound the expected gap at step *t*:

### Theorem 1

For every $t \ge 0$, we have that

$$\begin{aligned} \mathbb {E}[Gap(t)] = O(\sqrt{n} \log (n)). \end{aligned}$$

### Proof

From Lemma 2 we have that

$$\begin{aligned} \sum _{i=1}^n 2Gap_{A_{k}^i(t)}&\le \sum _{i=1}^n Gap_{A_{k-1}^i}(t)+\sum _{i=1}^n Gap_{A_{k-1}^{i+2^{m-k}}}(t) \\ {}&\quad \quad \quad \quad \quad \quad \quad + \sum _{i=1}^n 2 \max _{j \in A_{k}^i}|x_j(t) - x_{j+2^{m-k}}(t)| \\ {}&= 2\sum _{i=1}^n Gap_{A_{k-1}^i}(t)+2\sum _{i=1}^n \max _{j \in A_{k}^i}|x_j(t) - x_{j+2^{m-k}}(t)|. \end{aligned}$$

After dividing the above inequality by 2 and applying Lemma 3 we get that:

$$\begin{aligned} \sum _{i=1}^n Gap_{A_{k}^i}(t) \le \sum _{i=1}^n Gap_{A_{k-1}^i}(t)+\frac{n}{\sqrt{2^{m-k}}}\sqrt{\phi _{2^{m-k}}(t)}. \end{aligned}$$

Next, we sum up the above inequality for $k=1$ to *m*:

$$\begin{aligned} \sum _{k=1}^{m}\sum _{i=1}^n Gap_{A_{k}^i}(t) \le \sum _{k=1}^{m}\sum _{i=1}^n Gap_{A_{k-1}^i}(t)+\sum _{k=1}^{m} \frac{n}{\sqrt{2^{m-k}}}\sqrt{\phi _{2^{m-k}}(t)}. \end{aligned}$$

Recall that $\sum _{i=1}^n Gap_{A_{m}^i}(t)=n Gap(t)$ and $\sum _{i=1}^n Gap_{A_{0}^i}(t)=0$. Hence, we get that

$$\begin{aligned} n Gap(t) \le \sum _{k=1}^{m}\frac{n}{\sqrt{2^{m-k}}}\sqrt{\phi _{2^{m-k}}(t)}. \end{aligned}$$

Next, we apply Jensen’s inequality and Lemma 1:

$$\begin{aligned} n \mathbb {E}[Gap(t)]&\le \sum _{k=1}^{m}\frac{n}{\sqrt{2^{m-k}}}\mathbb {E}\sqrt{\phi _{2^{m-k}}(t)} \\&\le \sum _{k=1}^{m}\frac{n}{\sqrt{2^{m-k}}}\sqrt{\mathbb {E}[\phi _{2^{m-k}}(t)]} \\&\le \sum _{k=1}^{m}\frac{n}{\sqrt{2^{m-k}}} \sqrt{2^{m-k}(n-2^{m-k})} \\&\le m n\sqrt{n}=n(\log {n})\sqrt{n}. \end{aligned}$$

This gives us the proof of the theorem. $\square$

## Gap Lower Bound

Next we prove the following theorem, which lower bounds the second moment of the gap in expectation.

### Theorem 2

The following limit holds:

$$\begin{aligned} \lim _{t\rightarrow \infty } \mathbb {E}[Gap(t)^2]=\Omega (n\mathbb {E}[W^2])). \end{aligned}$$

### Proof

In this case we want to prove that not only does vector *Z*(*t*) have positive coordinates in expectation, but also $\mathbb {E}[z_{\lfloor \frac{n}{2}\rfloor }]$ converges to 0. This will give us that $\phi _{\lfloor \frac{n}{2}\rfloor }$ approaches its upper bound $(\lfloor \frac{n}{2}\rfloor \lceil \frac{n}{2}\rceil -1)\mathbb {E}[W^2]$ in expectation. Then, we can show that there exist two nodes (at distance $\lfloor \frac{n}{2}\rfloor$) such that the expected square of difference between their loads is $\Omega (n\mathbb {E}[w^2])$.

Recall from Eq. (11) that

$$\begin{aligned} n\mathbb {E}[z_{\lfloor \frac{n}{2} \rfloor (t+1)}]&= -\mathbb {E}[z_1(t)]+\mathbb {E}[z_{\lfloor \frac{n}{2}\rfloor +1}(t)] +\mathbb {E}[z_{\lfloor \frac{n}{2}\rfloor -1}(t)]+(n - 2)\mathbb {E}[z_{\lfloor \frac{n}{2}\rfloor }(t)]. \end{aligned}$$

We also know that Inequalities (12) hold for every *t*, hence we get that

$$\begin{aligned} \mathbb {E}[z_{\lfloor \frac{n}{2} \rfloor (t+1)}] \le \mathbb {E}[z_{\lfloor \frac{n}{2} \rfloor (t)}]-\frac{\mathbb {E}[z_1(t)]}{n}. \end{aligned}$$

The above inequality in combination with Inequalities (12) means that

17 $$\begin{aligned} \mathbb {E}[z_{\lfloor \frac{n}{2} \rfloor }(t+\lfloor \frac{n}{2} \rfloor +1)]&\le \mathbb {E}[z_{\lfloor \frac{n}{2} \rfloor }(t+1)]-\sum _{i=t}^{t+\lfloor \frac{n}{2} \rfloor } \frac{\mathbb {E}[z_1(i)]}{n} \nonumber \\ {}&\le \mathbb {E}[z_{\lfloor \frac{n}{2} \rfloor }(t+1)]- \frac{\mathbb {E}[z_1(t+\lfloor \frac{n}{2} \rfloor )]}{n} \end{aligned}$$

Again by using Eq. (11) and Inequalities (12), we can show that for every $1 \le i \le \lfloor \frac{n}{2} \rfloor -1$:

$$\begin{aligned} \mathbb {E}[z_i(t+1)] \ge \frac{\mathbb {E}[z_{i+1}(t)]}{n}. \end{aligned}$$

This gives us that:

$$\begin{aligned} \mathbb {E}[z_1(t+\lfloor \frac{n}{2} \rfloor )]&\ge \Big ( \frac{1}{n} \Big ) \mathbb {E}[z_2(t+\lfloor \frac{n}{2} \rfloor -1)] \ge \Big ( \frac{1}{n} \Big )^2 \mathbb {E}[z_3(t+\lfloor \frac{n}{2} \rfloor -2)] \\&\ge \cdots \\&\ge \Big ( \frac{1}{n} \Big )^{\lfloor \frac{n}{2}\rfloor - 1} \mathbb {E}[z_{\lfloor \frac{n}{2}\rfloor }(t+\lfloor \frac{n}{2}\rfloor - (\lfloor \frac{n}{2}\rfloor - 1))] \\ {}&= \Big ( \frac{1}{n} \Big )^{\lfloor \frac{n}{2}\rfloor - 1}\mathbb {E}[z_{\lfloor \frac{n}{2}\rfloor }(t+1)]. \end{aligned}$$

By plugging the above inequality in Inequality (17). we get that

$$\begin{aligned} \mathbb {E}[z_{\lfloor \frac{n}{2} \rfloor }&(t+\lfloor \frac{n}{2} \rfloor +1)] \le \mathbb {E}[z_{\lfloor \frac{n}{2} \rfloor }(t+1)]- \frac{\mathbb {E}[z_1(t+\lfloor \frac{n}{2} \rfloor )]}{n}\\&\le \mathbb {E}[z_{\lfloor \frac{n}{2} \rfloor }(t+1)] - \Big ( \frac{1}{n} \Big )^{\lfloor \frac{n}{2}\rfloor }\mathbb {E}[z_{\lfloor \frac{n}{2}\rfloor }(t+1)] \\ {}&= \Bigg (1-\Big (\frac{1}{n} \Big )^{\lfloor \frac{n}{2}\rfloor } \Bigg )\mathbb {E}[z_{\lfloor \frac{n}{2}\rfloor }(t+1)]. \end{aligned}$$

Because $\Bigg (1-\Big (\frac{1}{n} \Big )^{\lfloor \frac{n}{2}\rfloor } \Bigg ) < 1$ and does not depend on *t*, we get that

$$\begin{aligned} \lim _{t\rightarrow \infty }\mathbb {E}[z_{\lfloor \frac{n}{2}\rfloor }(t)]=0. \end{aligned}$$

This means that$\lim _{t\rightarrow \infty }\mathbb {E}[\phi _{\lfloor \frac{n}{2}\rfloor }(t)]=\Omega (n^2\mathbb {E}[W^2]).$

Let $Gap_{\lfloor \frac{n}{2}\rfloor }(t)=\max _{1\le i \le n}|x_i(t)-x_{i+\lfloor \frac{n}{2}\rfloor }(t)|$. Note that:

$$\begin{aligned} Gap(t)^2\ge Gap_{\lfloor \frac{n}{2}\rfloor }(t)^2 \ge \frac{\phi _{\lfloor \frac{n}{2}\rfloor }(t)}{n}. \end{aligned}$$

Hence

$$\begin{aligned} \lim _{t\rightarrow \infty } \mathbb {E}[Gap(t)^2]=\Omega (n\mathbb {E}[W^2]). \end{aligned}$$

Unfortunately we are not able to obtain the lower bound on the gap, since our approach uses the fact that the upper bounds on *k*-hop potentials are ’tight’. Since our potentials are quadratic, we are not able to derive any kind of lower bound for the gap itself. Intuitively, this will be an issue with any argument which uses convex potential. $\square$

## Upper Bound on the Gap, General Case

To prove Theorem 1 for the general case, we need to redefine our sets $A_k^{i}$. In order to do this, for each *k* we define $2^k$ dimensional vector $\Delta _k=( \delta _k^1, \delta _k^2, \ldots , \delta _k^{2^k} )$. For $k=0$, we have that $\Delta _k=(n)$. For $\lfloor \log {n} \rfloor \ge k >0$ we set $\Delta _k=(\alpha _k, \delta _{k-1}^1-\alpha _k, \alpha _k, \delta _{k-1}^2-\alpha _k, \ldots , \alpha _k, \delta _{k-1}^{2^{k-1}}-\alpha _k)$. where

$$\begin{aligned} \alpha _k= {\left\{ \begin{array}{ll} \lfloor \frac{n}{2^{k-1}} \rfloor /2,\quad {\text {if}} \lfloor \frac{n}{2^{k-1}} \rfloor \quad {\text {is even}}. \\ \Big \lfloor \lceil \frac{n}{2^{k-1}} \rceil /2 \Big \rfloor ,\quad {\text {otherwise}}.\\ \end{array}\right. } \end{aligned}$$

First we prove the following Lemma:

### Lemma 4

For any $\lfloor \log {n} \rfloor \ge k \ge 0$, we have that

1. $$\sum _{i=1}^{2^k} \delta _k^{i}=n$$.

2. For any $1 \le i \le 2^k$, $\delta _k^i \in \{ \lceil \frac{n}{2^k} \rceil \, \lfloor \frac{n}{2^k} \rfloor \}$ (notice that this means $\alpha _k=\lfloor \frac{n}{2^k}\rfloor$ or $\alpha _k=\lceil \frac{n}{2^k}\rceil$).

### Proof

We prove the lemma using induction on *k*. Base case $k=0$ holds trivially. For the induction step, assume that Properties 1 and 2 hold for $k-1$, we aim to prove that they hold for *k* as well. We have that $\sum _{i=1}^{2^k} \delta _k^{i}=\sum _{i=1}^{2^{k-1}} (\alpha _k+\delta _{k-1}^i-\alpha _k)=\sum _{i=1}^{2^{k-1}} \delta _{k-1}^i=n$. To prove Property 2 we consider several cases:

**Case 1** $\frac{n}{2^{k-1}} =2q$, for some integer *q*.

We have that $\alpha _k=q$, and hence for any $1 \le i \le 2^{k-1}$, $\delta _{k-1}^i-\alpha _k=q$. Since $\lfloor \frac{n}{2^k} \rfloor =q$, Property 2 holds.

**Case 2** $\frac{n}{2^{k-1}} =2q+1$, for some integer *q*.

We have that $\alpha _k=q$, and hence for any $1 \le i \le 2^{k-1}$, $\delta _{k-1}^i-\alpha _k=q+1$. Since $\lfloor \frac{n}{2^k} \rfloor =q$ and $\lceil \frac{n}{2^k} \rceil =q+1$, Property 2 holds.

**Case 3** $\frac{n}{2^{k-1}} =2q+\epsilon$, for some integer *q* and $0< \epsilon < 1$.

We have that $\lfloor \frac{n}{2^{k-1}} \rfloor =2q$ and $\lceil \frac{n}{2^{k-1}} \rceil =2q+1$. Additionally, $\alpha _k=q$, and hence for any $1 \le i \le 2^{k-1}$, $(\delta _{k-1}^i-\alpha _k) \in \{q, q+1\}$. Since $\lfloor \frac{n}{2^k} \rfloor =q$ and $\lceil \frac{n}{2^k} \rceil =q+1$, Property 2 holds.

**Case 4** $\frac{n}{2^{k-1}} =2q+1+\epsilon$, for some integer *q* and $0< \epsilon < 1$.

We have that $\lfloor \frac{n}{2^{k-1}} \rfloor =2q+1$ and $\lceil \frac{n}{2^{k-1}} \rceil =2q+2$. Additionally, $\alpha _k=q+1$, and hence for any $1 \le i \le 2^{k-1}$, $(\delta _{k-1}^i-\alpha _k) \in \{q, q+1\}$. Since $\lfloor \frac{n}{2^k} \rfloor =q$ and $\lceil \frac{n}{2^k} \rceil =q+1$, Property 2 holds. $\square$

Next, for $\lfloor \log {n} \rfloor \ge k >0$ we set

$$\begin{aligned} A_k^i=\{i, i+\delta _k^1, i+\delta _k^1+\delta _k^2, \ldots , i+\sum _{j=1}^{2^k-1} \delta _k^j\}. \end{aligned}$$

It is easy to see that for any $\lfloor \log {n} \rfloor \ge k >0$ and *i*, we have that $|A_{k}^i|=2^k$, $A_{k}^i=A_{k-1}^i \cup A_{k-1}^{i+\alpha _k}$ and $A_{k-1}^i \cap A_{k-1}^{i+\alpha _k}=\emptyset$. Also notice that for any $u \in A_{k-1}^i$, $u+\alpha _k \in A_{k-1}^{i+\alpha _k}$ and for any $u \in A_{k-1}^{i+\alpha _k}$, $u-\alpha _k \in A_{k-1}^{i}$.

Next we prove the lemma which is similar to the Lemma 2 for $n=2^m$ case:

### Lemma 5

For any $1 \le i \le n$ and $\lfloor \log {n} \rfloor \ge k > 0$, we have that

18 $$\begin{aligned} 2Gap_{A_{k}^i}(t) \le 2 \max _{j \in A_{k}^i}|x_j(t) - x_{j+\alpha _k}(t)| + Gap_{A_{k-1}^{i+\alpha _k}}(t)+ Gap_{A_{k-1}^i}(t). \end{aligned}$$

### Proof

Notice that the statement we need to prove is identical to that of Lemma 2, with the exception that in the letter we use $2^{m-k}$ instead of $\alpha ^k$. The proofs are also almost identical ($2^{m-k}$ can be simply replaced with $\alpha _k$). The only difference is that the proof of Lemma 2 uses the property that for any $u \in A_{k-1}^{i+2^{m-k}}$, $|x_u(t)-x_{u+2^{m-k}}(t)| \le \max _{j \in A_k^i} |x_j(t)-x_{j+2^{m-k}}(t)|$ and $u+2^{m-k} \in A_{k-1}^i$. Instead, we will use the property that for any $u \in A_{k-1}^{i+\alpha _k}$, $|x_u(t)-x_{u-\alpha _k}(t)| \le \max _{j \in A_k^i} |x_j(t)-x_{j+\alpha _k}(t)|$ and $u-\alpha _k \in A_{k-1}^i$. $\square$

Next, we upper bound $\sum _{i=1}^n \max _{j \in A_{k}^i}|x_j(t) - x_{j+\alpha _k}(t)|$, by proving the following lemma, which is the analogue of Lemma 3.

### Lemma 6

19 $$\begin{aligned} \sum _{i=1}^{n} \max _{j \in A_{k}^i}|x_j(t) - x_{j+\alpha _k}(t)| \le \Big \lceil \frac{n}{\lfloor \frac{n}{2^k} \rfloor } \Big \rceil \sqrt{\lfloor \frac{n}{2^k}\rfloor } \sqrt{\phi _{\alpha _k}(t)} \end{aligned}$$

### Proof

Notice that for any $1\le u \le n$ and the sets $A_k^u, A_k^{u+1}, \ldots , A_k^{u+\lfloor \frac{n}{2^k} \rfloor -1}$ are disjoint, because for any $1 \le j \le 2^k$, $\delta _k^j \ge \lfloor \frac{n}{2^k} \rfloor$ (this means that for any $1 \le i \le n$, distances between consecutive vertices in $A_k^i$ are at least $\lfloor \frac{n}{2^k} \rfloor$). Using this fact and Cauchy–Schwarz inequality we get that

$$\begin{aligned}&\sum _{i=u}^{u+\lfloor \frac{n}{2^k} \rfloor -1} \max _{j \in A_{k}^i}|x_j(t) - x_{j+\alpha _k}(t)| \\&\qquad \le \sqrt{\lfloor \frac{n}{2^k}\rfloor } \sqrt{\sum _{i=u}^{u+\lfloor \frac{n}{2^k} \rfloor -1} \max _{j \in A_{k}^{i}}|x_j(t) - x_{j+\alpha _k}(t)|^2} \\&\qquad \le \sqrt{\lfloor \frac{n}{2^k}\rfloor } \sqrt{\sum _{j=1}^n |x_j(t) - x_{j+\alpha _k}(t)|^2}=\sqrt{\lfloor \frac{n}{2^k}\rfloor } \sqrt{\phi _{\alpha _k}(t)} \end{aligned}$$

Since the above inequality holds for any *u* we can write that:

$$\begin{aligned} \sum _{i=1}^{n} \max _{j \in A_{k}^i}|x_j(t) - x_{j+\alpha _k}(t)| \le \Big \lceil \frac{n}{\lfloor \frac{n}{2^k} \rfloor } \Big \rceil \sqrt{\lfloor \frac{n}{2^k}\rfloor } \sqrt{\phi _{\alpha _k}(t)}. \quad \square \end{aligned}$$

$$\square $$

With the above three lemmas in place, we are ready to prove Theorem 1 for general *n*.

From Lemma 5 we have that

$$\begin{aligned} \sum _{i=1}^n 2Gap_{A_{k}^i(t)}&\le \sum _{i=1}^n Gap_{A_{k-1}^i}(t)+\sum _{i=1}^n Gap_{A_{k-1}^{i+\alpha _k}}(t)\\ {}&\quad \quad \quad + \sum _{i=1}^n 2 \max _{j \in A_{k}^i}|x_j(t) - x_{j+\alpha _k}(t)| \\ {}&= 2\sum _{i=1}^n Gap_{A_{k-1}^i}(t)+2\sum _{i=1}^n \max _{j \in A_{k}^i}|x_j(t) - x_{j+\alpha _k}(t)|. \end{aligned}$$

After dividing the above inequality by 2 and applying Lemma 6, we get that:

$$\begin{aligned} \sum _{i=1}^n Gap_{A_{k}^i}(t) \le \sum _{i=1}^n Gap_{A_{k-1}^i}(t)+\Big \lceil \frac{n}{\lfloor \frac{n}{2^k} \rfloor } \Big \rceil \sqrt{\lfloor \frac{n}{2^k}\rfloor } \sqrt{\phi _{\alpha _k}(t)}. \end{aligned}$$

Notice that for any *i*, $Gap_{A_0}^i(t)=0$. Hence, after summing up the above inequality for $k=1$ to $\lfloor \log {n} \rfloor$ we get that

$$\begin{aligned} \sum _{i=1}^n Gap_{A_{\lfloor \log {n} \rfloor }^i} (t) \le \sum _{k=1}^{\lfloor \log {n} \rfloor } \Big \lceil \frac{n}{\lfloor \frac{n}{2^k} \rfloor } \Big \rceil \sqrt{\lfloor \frac{n}{2^k}\rfloor } \sqrt{\phi _{\alpha _k}(t)}. \end{aligned}$$

Let $i'={{\,\mathrm{arg\,min}\,}}_i{Gap_{A_{\lfloor \log {n} \rfloor }^i} (t)}$. Notice that consecutive vertices in $A_{\lfloor \log {n} \rfloor }^{i'}$ are 1 or 2 edges apart, hence for any $1 \le i \le n$, either $i \in A_{\lfloor \log {n} \rfloor }^{i'}$ or $i+1 \in A_{\lfloor \log {n} \rfloor }^{i'}$. This gives us that

$$\begin{aligned} Gap(t)&\le Gap_{A_{\lfloor \log {n} \rfloor }^{i'}} (t)+2\max _{i} |x_i(t)-x_{i+1}(t)| \\&= Gap_{A_{\lfloor \log {n} \rfloor }^{i'}} (t)+2\sqrt{\max _{i} |x_i(t)-x_{i+1}(t)|^2} \le Gap_{A_{\lfloor \log {n} \rfloor }^{i'}} (t)+2\sqrt{\phi _1(t)}. \end{aligned}$$

By combining the above two inequalities we get that

$$\begin{aligned} n Gap(t)&\le n Gap_{A_{\lfloor \log {n} \rfloor }^{i'}} (t)+2n\sqrt{\phi _1(t)} \le \sum _{i=1}^n Gap_{A_{\lfloor \log {n} \rfloor }^i} (t)+2n\sqrt{\phi _1(t)} \\&\le \sum _{k=1}^{\lfloor \log {n} \rfloor } \Big \lceil \frac{n}{\lfloor \frac{n}{2^k} \rfloor } \Big \rceil \sqrt{\lfloor \frac{n}{2^k}\rfloor } \sqrt{\phi _{\alpha _k}(t)}+n\sqrt{\phi _1(t)}. \end{aligned}$$

Next, we apply Jensen’s inequality and Lemma 1 (we are going to use a looser upper bound: $\mathbb {E}[\phi _i(t)] \le i(n-i)-1 \le in)$

$$\begin{aligned} n \mathbb {E}[Gap(t)]&\le 2n \mathbb {E}\sqrt{[\phi _{1}(t)]}+\sum _{k=1}^{\lfloor \log {n} \rfloor } \Big \lceil \frac{n}{\lfloor \frac{n}{2^k} \rfloor } \Big \rceil \sqrt{\lfloor \frac{n}{2^k}\rfloor } \mathbb {E}\sqrt{\phi _{\alpha _k}(t)} \\&\le 2n\sqrt{\mathbb {E}[\phi _{1}(t)]}+\sum _{k=1}^{\lfloor \log {n} \rfloor } \Big \lceil \frac{n}{\lfloor \frac{n}{2^k} \rfloor } \Big \rceil \sqrt{\lfloor \frac{n}{2^k}\rfloor } \sqrt{\mathbb {E}[\phi _{\alpha _k}(t)]} \\&\le 2n\sqrt{n}+\sum _{k=1}^{\lfloor \log {n} \rfloor } \Big \lceil \frac{n}{\lfloor \frac{n}{2^k} \rfloor } \Big \rceil \sqrt{\lfloor \frac{n}{2^k}\rfloor } \sqrt{\alpha _k n} =O(n\sqrt{n}\log {n}). \end{aligned}$$

This completes the proof.

## Experimental Validation

**Fig. 1:**
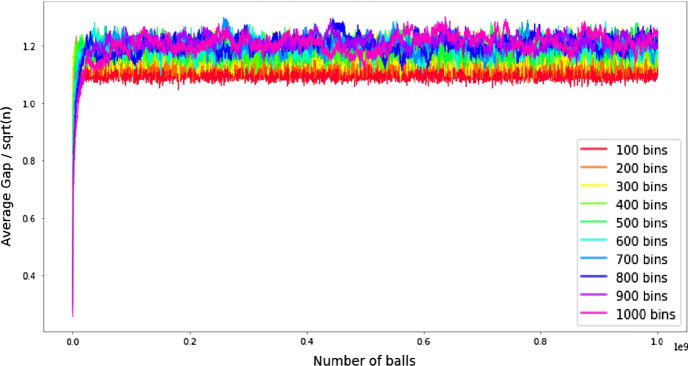
The evolution of average gap divided by square root of *n*, where *n* is the number of bins

On the practical side, we implemented our load balancing algorithm with unit weight increments on a cycle. The results confirm our hypothesis that the gap is of order $\Theta (\sqrt{n})$. In our experiment we observe the evolution of gap as we perform up to $10^9$ increment operations. In Fig. 1 we ran our experiment 100 times and calculated average gap over all runs. *x*-axis shows number of balls thrown (which is the same as the number of increments) and *y*-axis is current average gap divided by $\sqrt{n}$. The experiment shows that once the number of thrown balls is large enough, the gap stays between $\sqrt{n}$ and $1.4\sqrt{n}$.

## Harary Graph, Upper Bound on the Gap

Recall that the Harary graph $H_{k,n}$ is a *k*-connected graph with *n* vertices, which has the smallest possible number of edges. In this section we show that our approach can be extended to the Harary graph $H_{4,n}$ (unless specified we will assume $H_{4,n}$ to be the Harary graph): each vertex *i* is connected with edges to vertices $i-1$, $i+1$ (called cycle edges), $i-2$ and $i+2$ (called extra edges). As before, the operation consists of picking an edge u.a.r and doing increment and averaging (for the simplicity we assume that increments have unit weights, the result can be extended to the random weights, in the similar fashion to the cycle case). After careful calculations which mimic the calculations for the cycle case, but by taking extra edges of the Harary graph into the account, we can derive the following equations for the hop potentials (hop distance is counted over the cycle edges only):

$$\begin{aligned} {\left\{ \begin{array}{ll} \mathbb {E}[\phi _1(t+1)] = \frac{n-2}{n}\mathbb {E}[\phi _1(t)]+\frac{3}{4}+\frac{\mathbb {E}[\phi _1(t)]}{4n}+ \frac{\mathbb {E}[\phi _3(t)]}{2n}\\ \mathbb {E}[\phi _2(t+1)] = \frac{n-2}{n}\mathbb {E}[\phi _2(t)]+\frac{3}{4}-\frac{\mathbb {E}[\phi _2(t)]}{4n}+ \frac{\mathbb {E}[\phi _3(t)]}{2n}+\frac{\mathbb {E}[\phi _4(t)]}{2n}\\ \dots \\ \mathbb {E}[\phi _{k}(t+1)] = \frac{n-2}{n}\mathbb {E}[\phi _k(t)]+1-\frac{\mathbb {E}[\phi _1(t)]}{2n}-\frac{\mathbb {E}[\phi _2(t) ]}{2n}+ \frac{\mathbb {E}[\phi _{k-2}(t)]}{2n}+\frac{\mathbb {E}[\phi _{k-1}(t)]}{2n}\\ \quad \quad \quad \quad \quad \quad \quad \quad + \frac{\mathbb {E}[\phi _{k+1}(t)]}{2n}+ \frac{\mathbb {E}[\phi _{k+2}(t)]}{2n} \\ \end{array}\right. } \end{aligned}$$

Recall that for the cycle potential $\phi _k(t+1)$ depends on the potentials $\phi _k(t)$, $\phi _{k+1}(t)$, $\phi _{k-1}(t)$ and $\phi _1(t)$ (please see Eq. (8)). In the case of Harary graph $\phi _k(t+1)$ depends on the potentials $\phi _{k+2}(t)$, $\phi _{k+1}(t)$, $\phi _k(t)$, $\phi _{k-1}(t)$, $\phi _{k-2}(t)$, $\phi _1(t)$ and $\phi _2(t)$. The reason is that we are able to perform load balancing operation on two hop neighbours. Similarly, if we have a graph where each vertex is connected with all vertices which are at hop distance at most $\ell$ (this is the Harary graph $H_{2\ell ,n}$), then $\phi _k(t+1)$ will depend on $\phi _{k+\ell }(t)$, $\dots$, $\phi _{k+1}(t)$, $\phi _{k}(t)$, $\phi _{k-1}(t)$, $\dots$, $\phi _{k-\ell }(t)$, and $\phi _1(t)$, $\phi _2(t)$, $\dots$, $\phi _{\ell }(t)$. Next step is to find the stationary points for the hop potentials. That is: the values which stay the same after we apply step given by the above equations. As before (please see Lemma 1), these values will be used as the upper bounds for the expected values of potentials. In the case of $H_{4,n}$, we get that for every *t* and *k*:

20 $$\begin{aligned} \mathbb {E}[\phi _k(t)] \le \frac{2}{5}k(n-k)+\alpha . \end{aligned}$$

Here, extra term $\alpha$ has a closed form, which we omit and instead concentrate on the property that it is upper bounded by 2*n*. Observe that since the Harary graph contains cycle and we defined our hop potentials based on the hop counts of that cycle, we can upper bound the gap by using:

21 $$\begin{aligned} \mathbb {E}[Gap(t)] \le \sum _{k=1}^m \frac{1}{\sqrt{2^{m-k}}}\mathbb {E}\sqrt{\phi _{2^{m-k}}(t)} \le O(\sqrt{n}\log {n}). \end{aligned}$$

Notice that since $\alpha \le 2n$, for large enough *k* and *n*, the upper bound for $\mathbb {E}[\phi _k(t)]$ can be two times smaller than the upper bound for the cycle case, which was shown in Lemma 1. Hence, we can use this to slightly improve the constant hidden by big *O* notation in the upper bound. In general, we conjecture that for any pair of parameters $1 \le l_1 < l_2 \le n$, a gap for the Harary graph $H_{2l_2,n}$ is smaller than a gap for the Harary graph $H_{2l_1,n}$. Unfortunately, we are not able to provide the exact dependence of a gap on the Harary graph parameter.

## Discussion and Future Work

We have shown that in the case of dynamic averaging on a cycle the gap between highest and lowest loaded bins is upper bounded by $O(\sqrt{n}\log {n})$ in expectation. Additionally we showed that the expected square of the gap is lower bounded by $\Omega (n)$. It the future, it would be interesting to further tighten our results, matching our experimental analysis. We conjecture that the “correct” bound on the expected gap is $\Theta (\sqrt{n})$. As already discussed, we also plan to extend our results to more general graph families, in particular grid graphs.

### Comparison of Two-Choice and Averaging Load Balancing

Finally, it is interesting to ask if it is possible to extend our gap bounds to the case of the classic two-choice load balancing process. In particular, is it possible to show that the gap in the case of averaging process is always smaller in expectation than the gap in the case of two choice process? Intuitively this should be the case, since the load balancing operation in the case of averaging can be viewed as picking up a random edge, incrementing the load of the less loaded endpoint, and then averaging the values. The extra averaging step should not make the gap larger. Indeed, the exponential potential used to analyse the gap in [17] can be used to upper bound the gap for the averaging process, since the exponential function is convex and averaging values does not increase it (by Jensen’s inequality).

Unfortunately, it is not clear if averaging helps to actually *decrease* the exponential potential. Additionally, this argument shows that averaging does not make the gap worse if applied to the particular technique of upper bounding the gap, and it is not clear if the gap itself is actually smaller, if we use averaging on top of the two-choice process. We conjecture that there exists a majorization argument which is based on *how often* the process performs the averaging step. More precisely, we consider the setting where after the increment step (using two choice), we perform averaging with probability $\beta$. The gap should decrease in expectation as we increase $\beta$. Note that the only result which lower bounds the gap for the two-choice process on the cycle is the straightforward $\Omega (\log n)$ lower bound which can be shown for the clique [17]. Where The lower bound comes from the observation that if $\Omega (n \log n)$ balls of weight one are placed into *n* bins according to the two choice process, then the average load is $\Omega (\log n)$ and with constant probability there exists a bin which is empty. What would make the existence of the majorization argument interesting is that it would allow us to show that the lower bound we derived on the second moment of the gap while always performing averaging step on the cycle ($\beta =1$) can be automatically used as the lower bound on the gap for two choice on the cycle ($\beta =0$). We plan to investigate this connection in future work.
